# The Impact of Adverse Childhood Experiences on Nicotine Withdrawal Symptoms

**DOI:** 10.1007/s11469-025-01516-z

**Published:** 2025-06-26

**Authors:** Peyton Lehman, Ashley Petersen, Katherine Harrison, Sharon Allen

**Affiliations:** 1 University of Minnesota Medical School, Minneapolis, MN 55455 USA; 2 Division of Biostatistics and Health Data Science, University of Minnesota School of Public Health, Minneapolis, MN 55455 USA; 3 Department of Family Medicine and Community, Health, University of Minnesota Medical School, Minneapolis, MN 55455 USA; 4 St. Paul, USA

**Keywords:** Adverse childhood experiences (ACEs), Nicotine withdrawal, Tobacco cessation, Smoking reduction, Resilience

## Abstract

Adverse childhood experiences (ACEs) are a significant predictor of various physical and psychological problems in adulthood. This secondary analysis of a randomized smoking cessation trial examined the association between ACEs and the severity of nicotine withdrawal during smoking cessation. ACEs were assessed at baseline using a 10-item questionnaire, while nicotine withdrawal symptoms were measured weekly with the Minnesota Nicotine Withdrawal Scale (MNWS). No overall association was found between ACEs and MNWS craving scores across the entire sample. However, significant associations were observed when stratifying by smoking reduction versus cessation. Among those who reduced smoking, each additional ACE was linked to a 3% higher MNWS craving score. In contrast, among those who quit smoking, each additional ACE was associated with an 8% lower craving score. These findings suggest that resilience factors may influence the relationship between ACEs and tobacco dependence, warranting further investigation to improve cessation outcomes.

Adverse childhood experiences (ACEs), such as abuse, household drug use, or homelessness, are early life stressors that can disrupt normal development (Barnes et al., 2020). Past research studies have traced positive correlations between ACEs and future health effects, especially regarding chronic illness, risky behaviors, and addiction (Chang et al., 2019). The physiological stress response may contribute to long-term health effects through sustained activation of stress pathways (Anda et al., 2006; Sher et al., 2020). Moreover, exposure to adversity during the developmental age increases the likelihood of various mental and behavioral consequences, including conduct disorder, anxiety, depression, post-traumatic stress disorder, and substance abuse (Nelson et al., 2020). ACEs are disproportionately more prevalent in marginalized and low-income communities, making them a preventable problem of societal inequality and health disparity (Chung et al., 2016; Walsh et al., 2019).

Studies have repeatedly linked ACEs to increased tobacco use, earlier initiation, and higher nicotine dependence (Abufarsakh et al., 2024; Alcalá et al., 2016). One potential driver of this relationship between childhood adversity and initiation of substance use could lie within the family dynamic itself. A recent systematic review highlighted how different parental styles, specifically neglectful parenting, can directly influence the likelihood of adolescent substance use by hindering the development of beneficial coping mechanisms and increasing the probability of seeking comfort in substances (Shahzadi et al., 2023). Parental substance use may also be associated with an increased risk of adolescent substance use, and this relationship is further influenced by various moderating factors, including the childhood school environment, family income, and individual mental health (Rodríguez-Ruiz et al., 2025). Ultimately, multiple factors likely contribute to the higher rates of tobacco use among individuals who have experienced childhood adversity.

While increased adverse childhood experiences (ACEs) have been consistently linked to higher smoking rates, the relationship between childhood adversity and nicotine withdrawal remains less clear. The hypothalamic–pituitary–adrenal (HPA) axis plays a central role in regulating the body’s response to acute stressors. In a previous study, early-life adversity was associated with heightened HPA activity (al’Absi et al., 2017). Individuals with this dysregulated stress response were found to experience more severe nicotine withdrawal and higher relapse rates. Another possible contributor to variations in withdrawal severity is the genetic component of the stress response. Some individuals are genetically predisposed to stress-response abnormalities, which may increase their risk of intense withdrawal symptoms and relapse (Jensen & Sofuoglu, 2015). Although these patterns are not exclusive to those with a history of childhood adversity, the biological stress response remains a key factor in nicotine withdrawal. Both environmental and genetic factors likely influence the function of the HPA axis and partially explain why individuals may have different experiences when withdrawing from nicotine.

While ACEs have been linked to increased vulnerability, not all individuals respond the same way. Differences such as resilience or adaptive coping mechanisms may also influence how nicotine withdrawal is experienced. Exploring these variations could improve personalized approaches to smoking cessation.

This study aims to determine if ACEs are associated with the severity of subjective nicotine withdrawal and craving symptoms in current users attempting to quit smoking. We hypothesize that those who experienced more ACEs will tend to report more severe nicotine withdrawal and higher craving.

## Methods

This secondary analysis uses data from a randomized smoking cessation trial (Tosun et al., 2019). The parent study, which was IRB-approved by the University of Minnesota in December 2012, randomized 216 participants (113 males and 103 females) to placebo vs. progesterone (200 mg twice daily) for the 12-week study period of tobacco abstinence.

The parent study participants were selected based on the following criteria: women (ages 18–50 with regular menstrual cycles) and men (ages 18–60) who smoked at least five cigarettes per day (CPD) and were motivated to begin tobacco abstinence. Exclusion criteria included the use of other tobacco products, nicotine replacement therapy or cessation medications, use of recreational drugs (besides marijuana on a non-daily basis), the presence of psychosis, attention-deficit/hyperactivity disorder, bipolar disorder, or major depressive disorder, other substance dependence, unstable psychotropic medications (new to the participant within the last 3 months), exogenous hormone use, pregnant or breastfeeding (currently or within the last 3 months), or other contraindications to the study medication (exogenous progesterone). Participants were recruited in the Minneapolis/St. Paul metro area in Minnesota via pamphlets, flyers, and social media advertisements. All participants were financially compensated for their participation in the study (Tosun et al., 2019).

In the parent study, participants were assessed for relapse for 12 weeks following their assigned quit date, which was approximately 1 week after starting the study medication. Adherence to the study medication was confirmed with riboflavin within the capsule, which resulted in urine color change (Tosun et al., 2019). The study found a significant difference between the progesterone vs. placebo groups in the primary outcome, self-reported 7-day point prevalence abstinence (PPA) at week 4, among women but not men (Hughes et al., 2003). Abstinence was low among all groups, with the highest 7-day PPA at week 4 being 35.3% in females randomized to progesterone and the lowest being 17.3% in females randomized to placebo. Men in both groups had similar rates (progesterone, 23.2%; placebo, 21.1%).

In this secondary analysis, we focused on those with evidence of active quitting at week 4, given our interest in withdrawal and craving during quit attempts. We defined two study populations: (1) reduced smokers, those whose average self-reported CPD over the past 7 days at their week 4 visit was less than their baseline CPD, and (2) quitters, those who self-reported prolonged abstinence at week 4. Self-reported prolonged abstinence was defined as not having seven consecutive slips without 24 h between any two slips (Hughes et al., 2003).

Among the study measures collected in the parent study, we focused on ACEs and subjective nicotine withdrawal. ACEs were measured using a 10-item questionnaire where participants indicated which ACEs they experienced (e.g., separated/divorced parents, sexual abuse), and the final score was the number (range, 0–10) of ACEs experienced in the first 18 years of life with higher scores indicating increased exposure to trauma (Schulman & Maul, 2019). Subjective nicotine withdrawal symptoms were measured using two outcomes: the single item of craving and the withdrawal score from the Minnesota Withdrawal Scale (MNWS) (Hughes & Hatsukami, 1986, 1998). MNWS craving is captured via a single item (range, 0–4), and the MNWS withdrawal score is the average of seven other items (range, 0–4), where higher scores indicate more severe craving and withdrawal, respectively. The ACE questionnaire was collected at the screening visit, before randomization. The MNWS measures were captured at screening and baseline and then weekly throughout the intervention period (weeks 0–11).

Descriptive statistics were used to summarize the characteristics of the study populations, both overall and by the presence of ACEs (no ACEs vs. one or more ACEs). To estimate the associations between the number of ACEs self-reported at screening and the MNWS craving and withdrawal scores during active quitting, Poisson generalized estimating equations (GEE) models with a log link function were fit with an exchangeable working correlation to account for the weekly measurements (weeks 0–11) of the outcomes, MNWS craving and withdrawal scores. Models were adjusted for prolonged abstinence status, a sex-specific indicator for taking progesterone (given the sex-specific effect of progesterone observed in the parent study), week of the study (weeks 0–11), age, FTND score at screening, and CPD reported at baseline. Those randomized to the exogenous progesterone study group who had riboflavin-confirmed adherence at that week’s visit were considered to be taking progesterone for that week. Models were also fit with an interaction between prolonged abstinence status and the number of ACEs to test for a varying association of ACEs and MNWS scores between those who had prolonged abstinence vs. those who had only reduced their CPD. Lastly, the same model was fit, but only MNWS scores from weeks 4–11 (vs. weeks 0–11) were used to focus on only outcomes measured following the abstinence determination at week 4. GEE models were used to properly leverage the longitudinal outcome measures for increased statistical power. Given a sufficient number of clusters (participants), all GEE models were fit using robust standard errors, which are robust with respect to both the variance function and the within-subject correlation structure. No adjustments for multiple comparisons were performed. All analyses were performed in R version 4.2.1 (R Foundation for Statistical Computing, Vienna, Austria) with GEE models fit using the *geepack* R package (Højsgaard et al., 2006).

## Results

There were 140 participants (70 males and 70 females) known to have quit or reduced their smoking at week 4. Of these, 36 participants (16 males and 20 females) had prolonged abstinence at week 4. The percentage who reported experiencing one or more ACEs was 63% (66/104) in those who reduced their CPD at week 4 (relative to baseline) and 67% (24/36) in those who had prolonged abstinence at week 4. The characteristics of the participants who reduced or quit smoking are summarized in Tables 1 and 2, respectively.

**Table 1:** Summary of study population characteristics of those who reduced smoking

Characteristic	Overall, *N* = 140	No adverse childhood events, *N* = 50	At least one adverse childhood event, *N* = 90
Male	70 (50%)	27 (54%)	43 (48%)
Age (years)	38 (31, 45)	36 (30, 42)	38 (33, 45)
Race			
Black or African American	37 (26%)	13 (26%)	24 (27%)
Other	22 (16%)	6 (12%)	16 (18%)
White	81 (58%)	31 (62%)	50 (56%)
Education			
High school or less	39 (28%)	15 (30%)	24 (27%)
Some college/2-year degree	66 (47%)	18 (36%)	48 (53%)
College graduate or more	35 (25%)	17 (34%)	18 (20%)
Household income			
Less than $15,000	45 (33%)	19 (38%)	26 (30%)
$15,001 to $20,000	14 (10%)	3 (6.0%)	11 (12%)
$20,001 to $30,000	19 (14%)	5 (10%)	14 (16%)
$30,001 to $45,000	21 (15%)	6 (12%)	15 (17%)
$45,001 to $60,000	15 (11%)	7 (14%)	8 (9.1%)
$60,001 to $75,000	11 (8.0%)	4 (8.0%)	7 (8.0%)
More than $75,001	13 (9.4%)	6 (12%)	7 (8.0%)
Marital status			
Never married	86 (61%)	35 (70%)	51 (57%)
Currently married	26 (19%)	10 (20%)	16 (18%)
No longer married	28 (20%)	5 (10%)	23 (26%)
Cigarettes per day	12 (10, 17)	12 (10, 15)	12 (10, 17)
Fagerstrom Test for nicotine dependence	4 (3, 6)	4 (3, 6)	5 (3, 6)
Age started smoking (years)	17 (15, 18)	17 (15, 18)	16 (15, 19)
Quit attempts (number)	4 (2, 8)	4 (2, 8)	4 (2, 9)

**Table 2:** Summary of study population characteristics of those who quit smoking

Characteristic	Overall, *N* = 36	No adverse childhood events, *N* = 12	At least one adverse childhood event, *N* = 24
Male	16 (44%)	7 (58%)	9 (38%)
Age (years)	36 (30, 40)	36 (29, 40)	36 (32, 40)
Race			
Black or African American	8 (22%)	2 (17%)	6 (25%)
Other	7 (19%)	1 (8.3%)	6 (25%)
White	21 (58%)	9 (75%)	12 (50%)
Education			
High school or less	8 (22%)	1 (8.3%)	7 (29%)
Some college/2-year degree	14 (39%)	4 (33%)	10 (42%)
College graduate or more	14 (39%)	7 (58%)	7 (29%)
Household income			
Less than $15,000	13 (37%)	4 (33%)	9 (39%)
$15,001 to $20,000	1 (2.9%)	1 (8.3%)	0 (0%)
$20,001 to $30,000	5 (14%)	1 (8.3%)	4 (17%)
$30,001 to $45,000	8 (23%)	3 (25%)	5 (22%)
$45,001 to $60,000	4 (11%)	2 (17%)	2 (8.7%)
$60,001 to $75,000	2 (5.7%)	1 (8.3%)	1 (4.3%)
More than $75,001	2 (5.7%)	0 (0%)	2 (8.7%)
Marital status			
Never married	25 (69%)	8 (67%)	17 (71%)
Currently married	6 (17%)	4 (33%)	2 (8.3%)
No longer married	5 (14%)	0 (0%)	5 (21%)
Cigarettes per day	10 (8, 15)	11 (8, 14)	10 (8, 15)
Fagerstrom Test for nicotine dependence	4 (2, 6)	4 (3, 5)	4 (1, 6)
Age started smoking (years)	17 (16, 20)	18 (17, 20)	16 (16, 20)
Quit attempts (number)	4 (3, 10)	4 (2, 8)	5 (3, 10)

**Fig. 1:**
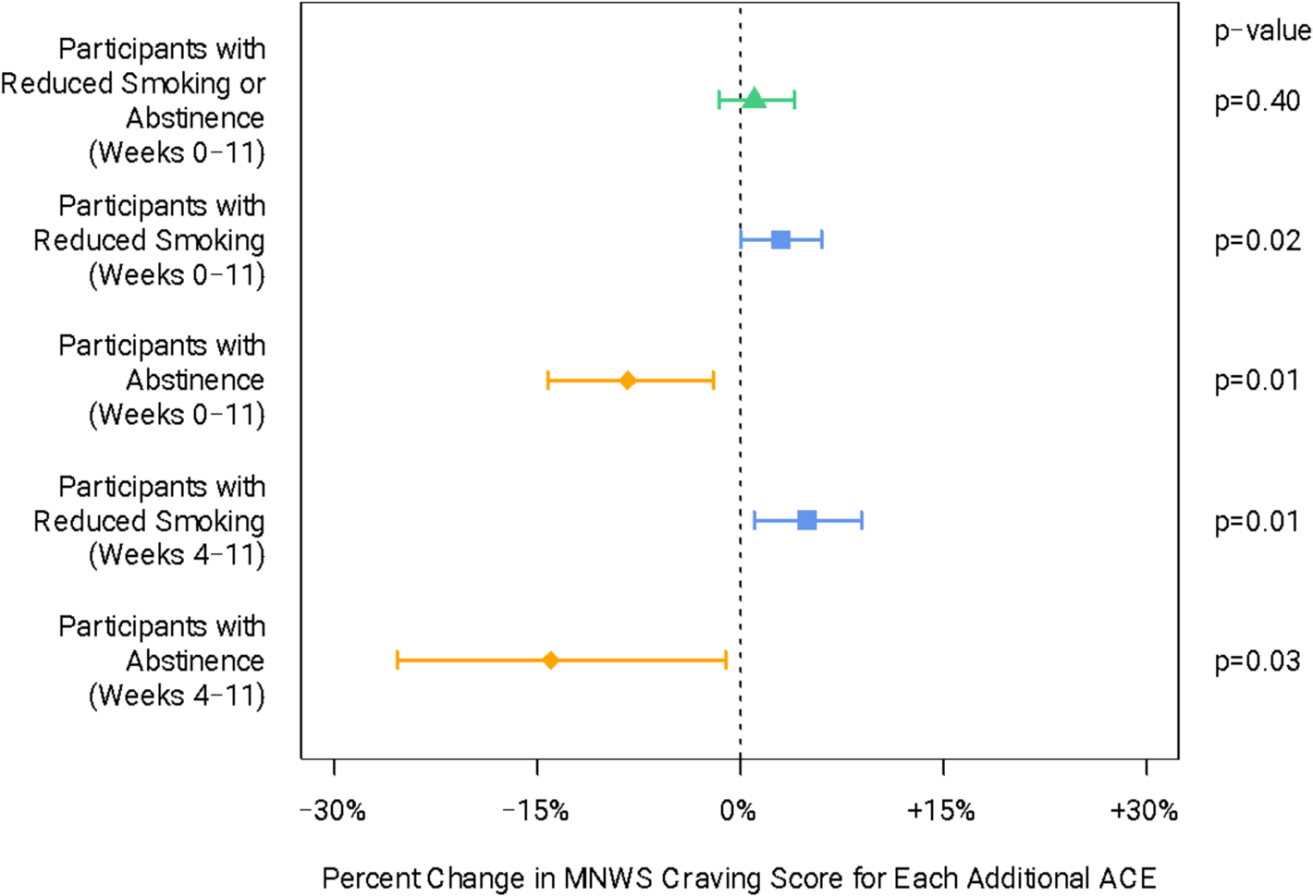
Associations between the number of adverse childhood experiences (ACEs) at screening and Minnesota Nicotine Withdrawal Scale (MNWS) craving scores collected over time (weeks 0–11 or weeks 4–11) among those who reduced their cigarettes per day and/or had prolonged abstinence at week 4

Figure 1 summarizes the associations between the number of ACEs at screening and the MNWS craving score during active quitting. We found no association between the number of ACEs and the MNWS craving score among all participants with reduced smoking or abstinence at week 4 (*p* = 0.40). However, there was a significant association when allowing the association to differ between those with reduced smoking vs. prolonged abstinence. Specifically, among those who reduced their smoking, each additional ACE was associated with a 3% higher MNWS craving score on average (95% confidence interval [CI], 0.4–6% higher; *p* = 0.02). Whereas, in those with prolonged abstinence, each additional ACE was associated with an 8% lower MNWS craving score on average (95% CI, 2–14% lower; *p* = 0.01). These associations remained when restricting the MNWS craving outcomes to the weeks following the abstinence determination (weeks 4–11) rather than considering all study weeks (weeks 0–11).

**Fig. 2:**
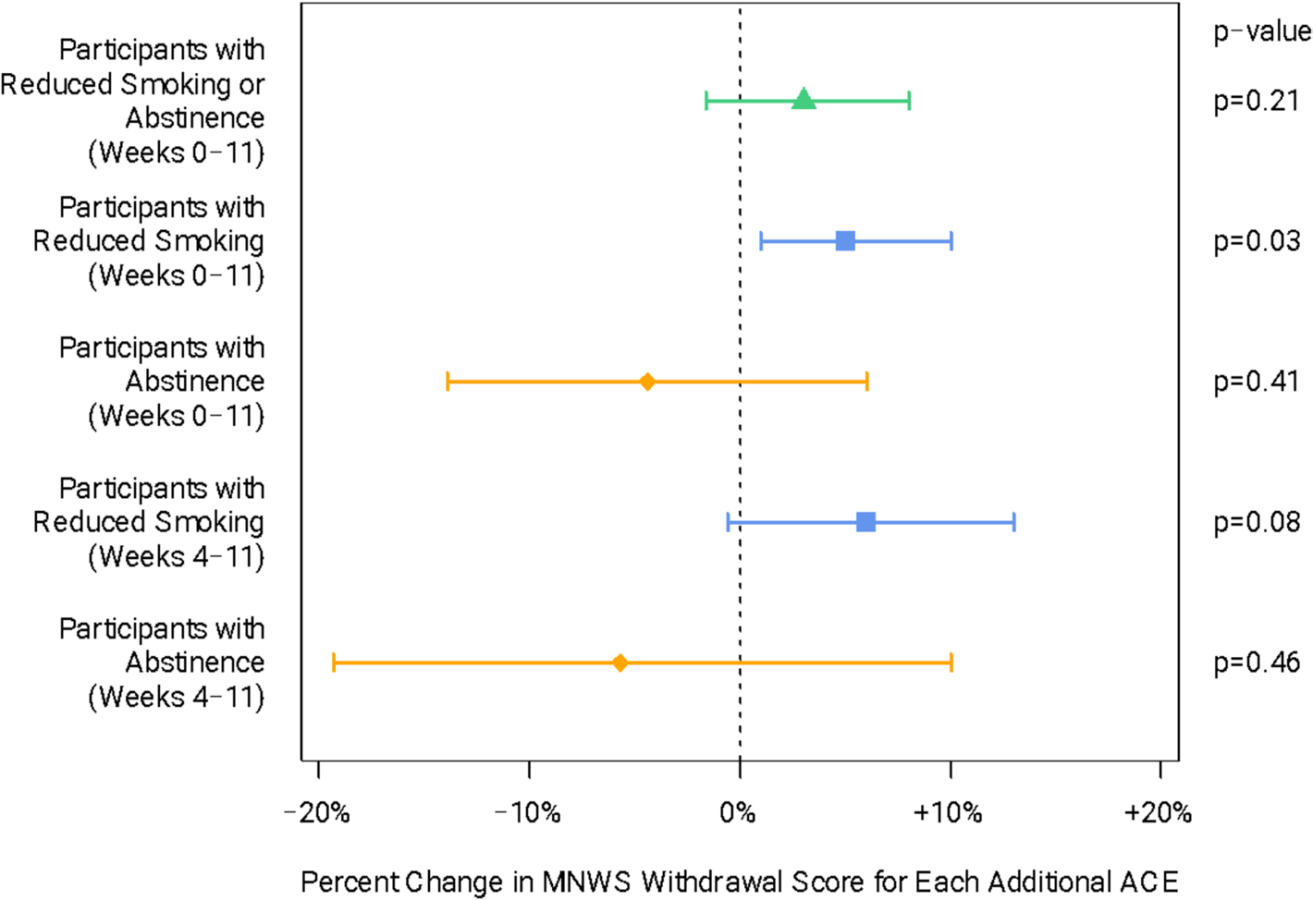
Associations between the number of adverse childhood experiences (ACEs) at screening and Minnesota Nicotine Withdrawal Scale (MNWS) withdrawal scores collected over time (weeks 0–11 or weeks 4–11) among those who reduced their cigarettes per day and/or had prolonged abstinence at week 4

Figure 2 summarizes the associations between the number of ACEs at screening and the MNWS withdrawal score during active quitting. Similar to MNWS craving, we found no association between the number of ACEs and the MNWS withdrawal score when considering all participants who quit or reduced their smoking at week 4 (*p* = 0.21). When allowing the association to differ between those who reduced vs. quit smoking, we found no association among participants who quit. However, there was a significant association for those who had only reduced their smoking but not quit with each additional ACE associated with, on average, a 5% higher MNWS withdrawal score (95% CI, 1–10% higher; *p* = 0.03). The association remained consistent when restricting the MNWS withdrawal score to only weeks 4–11, although it was no longer statistically significant (*p* = 0.08).

## Discussion

These study results offer insight into the complex relationship between nicotine withdrawal and the number of ACEs within an individual’s childhood. In the smoking reduction group, there was a correlation between increasing ACEs and higher withdrawal and craving scores. This finding aligns with the expected outcome of the study since past research has shown higher rates of tobacco initiation and tobacco addiction among individuals with more childhood adversity (Abufarsakh et al., 2024; Alcalá et al., 2016). In addition, the role of endogenous endocrine hormones in the pathophysiology of nicotine withdrawal and craving should be considered. Cortisol, for example, is a well-known hormone that modulates the physiologic stress response and has been previously found to correlate with tobacco withdrawal symptoms during smoking abstinence (Gourlay et al., 2004). These endocrine stress markers are elevated at baseline in those with higher childhood adversity, and this relationship potentially influences the individual experience of withdrawal and craving symptoms (al’Absi et al., 2017; Bolin et al., 2022).

Beyond physiological mechanisms, psychological factors may also mediate the ACE-withdrawal relationship. Past research has shown that childhood adversity is linked to a higher likelihood of dissociation and maladaptive coping behaviors in adulthood, which may contribute to addiction (Lyssenko et al., 2018; Wagner-Skacel et al., 2022). Dissociation, the process of disconnecting the integration of self from physical, emotional, and mental processing to tolerate distressing events, has been associated with substance use disorder. If these maladaptive coping mechanisms are indeed linked to ACEs and nicotine withdrawal, addressing them could serve as an intervention point for improved smoking cessation outcomes.

Interestingly, an association between increasing ACEs and lower craving scores was observed among individuals in the prolonged abstinence group. These contrasting results suggest that an underlying factor may influence resilience and the ability to cope with smoking abstinence in the prolonged abstinence group compared to the smoking reduction group. Specifically, the study data showed that among individuals who successfully fully abstained from smoking, those with more childhood adversity were more likely to report less severe withdrawal. In some individuals, exposure to childhood adversity, as well as other demographic stressors such as low socioeconomic status, may have forced them to develop resilience mechanisms that help them manage stress and cravings during nicotine abstinence later in life. A previous meta-analysis has shed light on this pattern in the context of depression. While trauma and depression are significantly correlated, resilience has been found to mediate this association (Watters et al., 2021).

The findings of this study highlight the importance of employing a trauma-informed approach when managing substance dependence in any patient population. Recognizing how childhood trauma could manifest as maladaptive coping mechanisms and low resilience presents a possible area of intervention for addiction specialists, psychiatrists, and primary care providers. ACEs represent a current public health crisis that can be addressed in an outpatient setting through widespread screening (Dube, 2018). In doing so, providers are better able to understand the trauma driving the behaviors of their patients. Patients attempting to pursue smoking cessation may benefit immensely from psychiatric referral to build strong coping mechanisms and increase resilience, therefore increasing the likelihood of success.

Furthermore, future research could benefit from the integration of models such as the Resilience Scale for Adults (Friborg et al., 2003) and the Brief COPE Inventory (Rodrigues et al., 2022) to better quantify the complex relationship between trauma, resilience, coping mechanisms, and nicotine withdrawal.

While the findings of this study may have beneficial implications for patient-centered and trauma-informed care, certain limitations should be considered when interpreting the data. First, caution should be taken when generalizing the results, as our sample was limited to a specific geographic area, and the socioeconomic, racial, age, and gender distributions may not resemble those of the broader population. Another limitation relates to the use of self-reported measures in data collection, which may lead to recall bias or social desirability bias. For example, participants may have over- or under-reported ACEs based on their interpretation of how they may be perceived, which would skew the data. Finally, there are potential confounding variables that may influence the study's findings, including underlying mental health conditions and socioeconomic status. While the data from this present study originates from a randomized trial, this secondary analysis focusing on differences based on participants’ childhood adversity is observational. There may be residual confounding stemming from differences between those who experienced different numbers of ACEs. Many of the social determinants of health have been identified as predictors of nicotine use, and many of our study participants likely have external influences on their health and behavior (Assari et al., 2024). Ultimately, this study suggests that characteristics based on childhood experience may play a role in withdrawal from nicotine, and further research should be conducted to understand this relationship.

## Conclusion

This secondary analysis of a tobacco cessation trial explored the possible link between the amount of childhood adversity faced in one’s lifetime and the subjective severity of nicotine withdrawal in smoking abstinence. The findings of this work showed that the correlation between ACEs and tobacco craving varies based on complete smoking abstinence vs. smoking reduction, bringing to light the possibility of an extraneous variable related to coping mechanisms and resilience in influencing tobacco dependence. Future studies could expand upon the growing body of knowledge contributing to an evolving era of individualized medicine to improve tobacco cessation interventions.

## Data Availability

The data that support the findings of this study are available from the corresponding author upon reasonable request.
